# Stage-Specific and Selective Delivery of Caged Azidosugars into the Intracellular Parasite *Toxoplasma gondii* by Using an Esterase-Ester Pair Technique

**DOI:** 10.1128/mSphere.00142-19

**Published:** 2019-05-29

**Authors:** Tadakimi Tomita, Hua Wang, Peng Wu, Louis M. Weiss

**Affiliations:** a Department of Pathology, Albert Einstein College of Medicine, New York, New York, USA; b Department of Chemical Physiology, The Scripps Research Institute, La Jolla, California, USA; c Department of Medicine, Albert Einstein College of Medicine, New York, New York, USA; Indiana University School of Medicine

**Keywords:** chemical biology, click chemistry, glycobiology, intracellular pathogen, porcine esterase, small molecular delivery, *Toxoplasma gondii*

## Abstract

Toxoplasma gondii is an obligate intracellular parasite that chronically infects up to a third of the human population. The parasites persist in the form of cysts in the central nervous system and serve as a reservoir for the reactivation of toxoplasmic encephalitis. The cyst wall is known to have abundant *O*-linked *N*-acetylgalactosamine glycans, but the existing metabolic labeling methods do not allow selective labeling of intracellular parasite glycoproteins without labeling of host glycans. In this study, we have integrated Cu(I)-catalyzed bioorthogonal click chemistry with a specific esterase-ester pair system in order to selectively deliver azidosugars to the intracellular parasites. We demonstrated that α-cyclopropyl modified GalNAz was cleaved by porcine liver esterase produced in the parasites but not in the host cells. Our proof-of-concept study demonstrates the feasibility and potential of this esterase-ester click chemistry approach for the selective delivery of small molecules in a stage-specific manner.

**IMPORTANCE** Selective delivery of small molecules into intracellular parasites is particularly problematic due to the presence of multiple membranes and surrounding host cells. We have devised a method that can deliver caged molecules into an intracellular parasite, Toxoplasma gondii, that express an uncaging enzyme in a stage-specific manner without affecting host cell biology. This system provides a valuable tool for studying many intracellular parasites.

## Abstract (precis)

Selective delivery of small molecules into intracellular parasites is particularly problematic due to the presence of multiple membranes and surrounding host cells. We have devised a method that can deliver caged molecules into an intracellular parasite, Toxoplasma gondii, that express an uncaging enzyme in a stage-specific manner without affecting host cell biology. This system provides a valuable tool for studying many intracellular parasites.

## INTRODUCTION

Toxoplasma gondii is a ubiquitous protozoan parasite that is present as a latent (i.e., chronic) infection in approximately a third of the human population (1). When T. gondii infects a host, it proliferates as rapidly growing tachyzoites, resulting in mild flu-like symptoms. Following acute infection, this parasite differentiates into bradyzoites forming latent tissue cysts in the central nervous system (CNS) and muscles. These tissue cysts may persist for the lifetime of the host. Currently, there are no drugs that can successfully eradicate these latent T. gondii cysts (2). The cysts serve as a reservoir for the reactivation of toxoplasmosis when a host becomes immunocompromised, which can result in a life-threatening encephalitis in AIDS patients and organ transplant recipients (3). Under the parasitophorous vacuole membrane, these intracellular tissue cysts are covered by a highly glycosylated granular layer termed the cyst wall. This structure is critical for persistence of the cysts during latent infection (4). Binding of the cyst wall by various GalNAc glycan-binding lectins, e.g., Dolichos biflorus lectin (DBA), Vicia villosa lectin (VVA), Helix pomatia lectin (HPA), and Jacalin, as well as periodic acid-Schiff stain, has suggested the presence of *O*-GalNAc modifications on cyst wall proteins. A comprehensive glycomics study has validated the presence of *O*-linked GalNAc glycans by mass spectrometry analysis of sugars released by β-elimination of glycoproteins, specifically the presence of the core 5 structure (GalNAcα1,3GalNAcα1-Ser/Thr) in tachyzoites (5).

Previous attempts to identify the composition of the *O*-GalNAc glycoproteome using the *O*-GalNAc-binding lectins Jacalin (6) and VVA (7) resulted in the discovery of various potential glycoproteins. The procedures utilized for these studies involved purification of parasites that were lysed out (i.e., isolated) from their host cells. This eliminates the overwhelming amount of contaminating host glycoproteins; however, it also eliminates the parasite cyst wall glycoproteins, since these are components of the parasitophorous vacuole membrane, which is lost during the purification process. Thus, these lectin affinity purifications do not provide an assessment of the cyst wall glycoproteome.

Newer and more specific approaches to investigate glycoproteomes, such as bioorthogonal click chemistry, have been widely used in other systems to identify glycoproteins. In this method, organisms incorporate azidosugars into their glycoproteins, which are then selectively retrieved after click chemistry. This system had been used to examine GlcNAz-labeled glycoproteome in T. gondii successfully (8). However, a significant limitation of click chemistry is that azidosugars can enter host cell, e.g., cross a single membrane, but cannot pass across the intracellular parasite plasma membrane. Typically, alcohol groups of azidosugars are acetylated to increase membrane permeability. Once the azidosugar crosses the host cell plasma membrane, its acetyl groups are cleaved by endogenous esterases in the host cell cytosol, which renders the azidosugar nonpermeable to the plasma membrane of intracellular parasites. Therefore, current azidosugar labeling of T. gondii has to be performed in extracellular parasites. This puts these obligate intracellular parasites under stress conditions during the time of incorporation of the azidosugars and eliminates labeling of proteins in the cyst wall and matrix, since they are not generated by these extracellular parasites. To study the cyst wall and matrix, newer approaches are needed.

**FIG 1:**
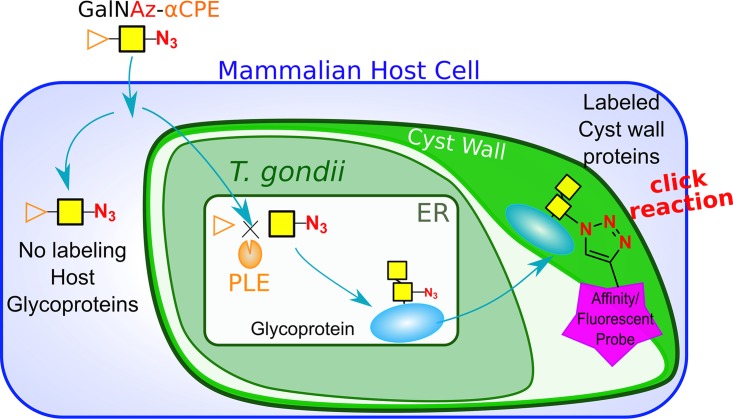
Schematic of selective sugar delivery using the esterase-ester pair click chemistry. The yellow square represents GalNAz, and the orange triangle represents the α-cyclopropyl ester caging group.

Our goal was to develop a system that could label parasite glycoproteins without labeling host glycoproteins. To confer this parasite specificity, we integrated the widely used bioorthogonal click chemistry with a caged substrate and a genetically encoded uncaging enzyme pair (Fig. 1). In recent years, several of those pair systems have been described, including a mutant cytochrome P450 monooxygenase-propagylic ether pair system (9), an artificial metalloenzyme-caged hormone pair system (10), and an esterase-ester pair system (11). We selected the esterase-ester pair system for our purpose. In this method, small molecules are caged with a specific bulky and inert ester-caging group, rendering the small molecule inaccessible to cellular targets unless the bulky group is removed. The caged molecule can only become uncaged by the enzyme porcine liver esterase (PLE). PLE is a carboxylesterase (EC 3.1.1.1) that hydrolyzes a broad range of carboxylic esters into an alcohol and a carboxylate (12). By selectively expressing PLE in a particular cell, this esterase-ester system was demonstrated *in vitro* to selectively target the cell cycle inhibitor monastrol to cells expressing PLE (11). This system has also been used *in vivo* to selectively inhibit NMDA receptors on neurons selectively by expressing PLE under a neuron-specific promoter treated with a caged NMDA-R inhibitor (13).

To take advantage of the esterase-ester specificity, we synthesized various azidosugars caged with the bulky ester group that prevented the incorporation of the azidosugar into glycans unless the ester protecting group was cleaved by PLE. We generated a T. gondii strain that heterologously expressed PLE with a T. gondii endoplasmic reticulum (ER) retention signal. The caged azidosugars were selectively cleaved in PLE-expressing parasites and incorporated, while the surrounding host cell had minimal signal due to the presence of the bulky caging group. This simple and robust system can be widely applied to other small molecules, such as other metabolic substrates or small molecule inhibitors used in a mixture of heterogeneous cell types or any genetically tractable intracellular parasites.

## RESULTS

### Heterologous expression of PLE in *T. gondii*.

**FIG 2:**
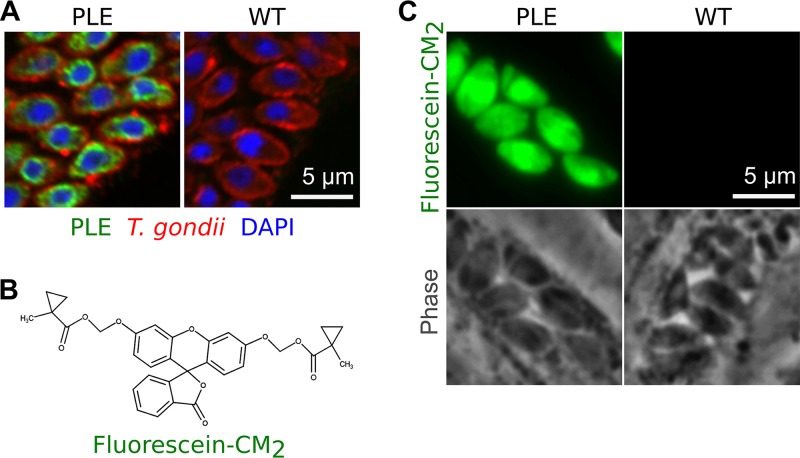
Porcine liver esterase (PLE) in T. gondii is functional and specific. (A) IFA image of HFFs infected with intracellular T. gondii (polyclonal anti-toxoplasma, red) expressing PLE (anti-FLAG, green). Heterologous expression of PLE is tolerated by T. gondii. (B) Molecular structure of caged fluorescein FITC-CM_2_, which is nonfluorescent unless it is uncaged. Measurement of fluorescence serves as a surrogate of PLE activity. (C) Epifluorescent live image of intracellular T. gondii incubated with FITC-CM_2_. Uncaging only happens in PLE-expressing parasites but not in host cell or wild-type (WT) parasites.

To determine whether the esterase-ester system was compatible with the parasite, PLE was heterologously expressed in T. gondii. The original ER retention signal was replaced with that of T. gondii BiP (14) for correct localization; a FLAG epitope tag sequence was inserted after a signal peptide for visualization of PLE. A strong constitutive promoter from the T. gondii GRA1 gene drove the expression of PLE. Figure 2A is immunofluorescence images of intracellular T. gondii probed with anti-FLAG antibody, and this demonstrates that overexpression PLE in parasite was tolerated by the parasite.

### Specific esterase activity of PLE in *T. gondii*.

To determine whether the expressed mammalian esterase PLE was functional in T. gondii, the ability of T. gondii expressing PLE to uncage a cyclopropyl ester-caged fluorescein (FITC-CM_2_; Fig. 2B) was investigated. FITC-CM_2_ has two cyclopropyl ester caging groups that render this FITC derivative nonfluorescent. Upon successful cleavage of the cyclopropyl ester caging group, FITC restores fluorescence; thus, it can serve as surrogate for esterase activity of PLE. Figure 2C is an image of live intracellular parasites that had been incubated with FITC-CM_2_ for 30 min. PLE-expressing parasites display bright green fluorescence, but wild-type parasites and host cells have no fluorescent signal. This demonstrates the cyclopropyl ester is specifically cleaved by PLE but is not cleaved by endogenous esterases in either the parasite or its host cells. This also verifies that the mammalian esterase PLE is functional when expressed in the intracellular parasite T. gondii.

### Parasite-specific sugar labeling by PLE.

**FIG 3:**
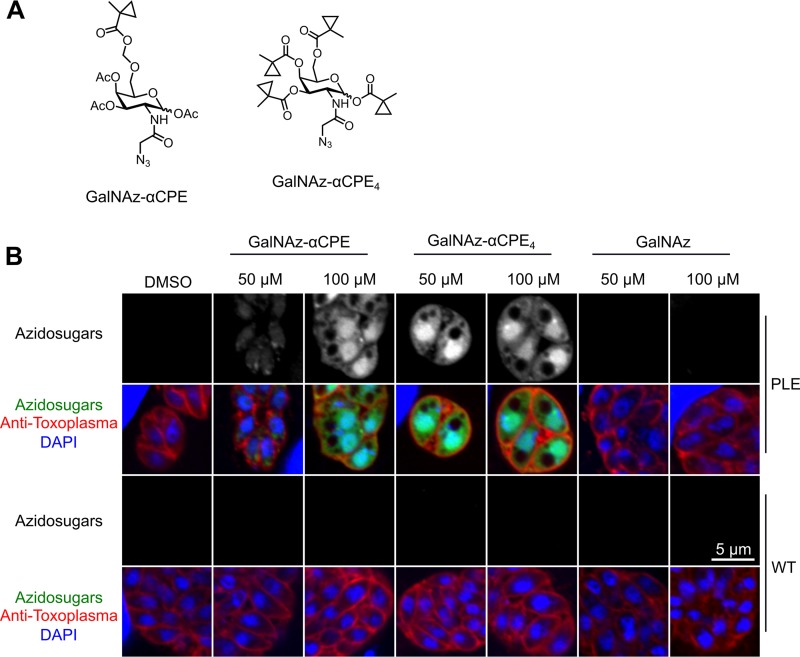
Selective sugar labeling with esterase-ester click chemistry. (A) Molecular structures of caged azidosugars. Ac, acetyl group. (B) Confocal fluorescent image of specific sugar labeling of intracellular parasites incubated with caged azidosugars. Both sugars are specifically uncaged only in the PLE-expressing T. gondii.

To determine the feasibility of combining this esterase-ester system with bioorthogonal click chemistry for parasite specific sugar labeling, caged azidosugars were synthesized. We were interested in the labeling of *O*-GalNAc glycans; therefore, hydroxyl groups of azide-modified GalNAc (GalNAz) were substituted with cyclopropyl esters. Figure 3A shows the chemical structures of the two sugars, GalNAz-αCPE and GalNAz-αCPE_4_. One has a substitution with a longer ester linkage (GalNAz-αCPE); the other has four substitutions with shorter linkages (GalNAz-αCPE_4_). All other hydroxyl groups are substituted with acetyl groups in order to increase permeability of the sugar across a plasma membrane. Detailed chemical synthesis protocols for these compounds are described in Text S1 in the supplemental material. Figure 3B demonstrates intracellular tachyzoites cultured in the presence of these azidosugars. The azide moiety was visualized by click chemistry with a fluorescent probe. While the wild-type parasites show minimal signal, PLE-expressing parasites have a bright signal in the nucleus, cytosol, and parasitophorous vacuole space. This indicates that the caged azidosugars were selectively uncaged in PLE-expressing parasites and then probably incorporated into parasite glycans. The azide signal was detected after fixation, membrane permeabilization, and the click reaction; therefore, only liberated sugar that was incorporated into macromolecules is likely detected. Upon examination, some of the vacuoles containing PLE-expressing parasites have extracellular signal within the parasitophorous vacuole membrane and matrix that suggests incorporation of azidosugars into the cyst wall (parasitophorous vacuole membrane) glycoproteins that have *O*-GalNAc glycans.

10.1128/mSphere.00142-19.1 TEXT S1 Synthesis of caged azidosugars. Download Text S1, PDF file, 0.2 MB. Copyright © 2019 Tomita et al. 2019 Tomita et al. This content is distributed under the terms of the Creative Commons Attribution 4.0 International license.

### Stage-specific azidosugar delivery by PLE.

**FIG 4:**
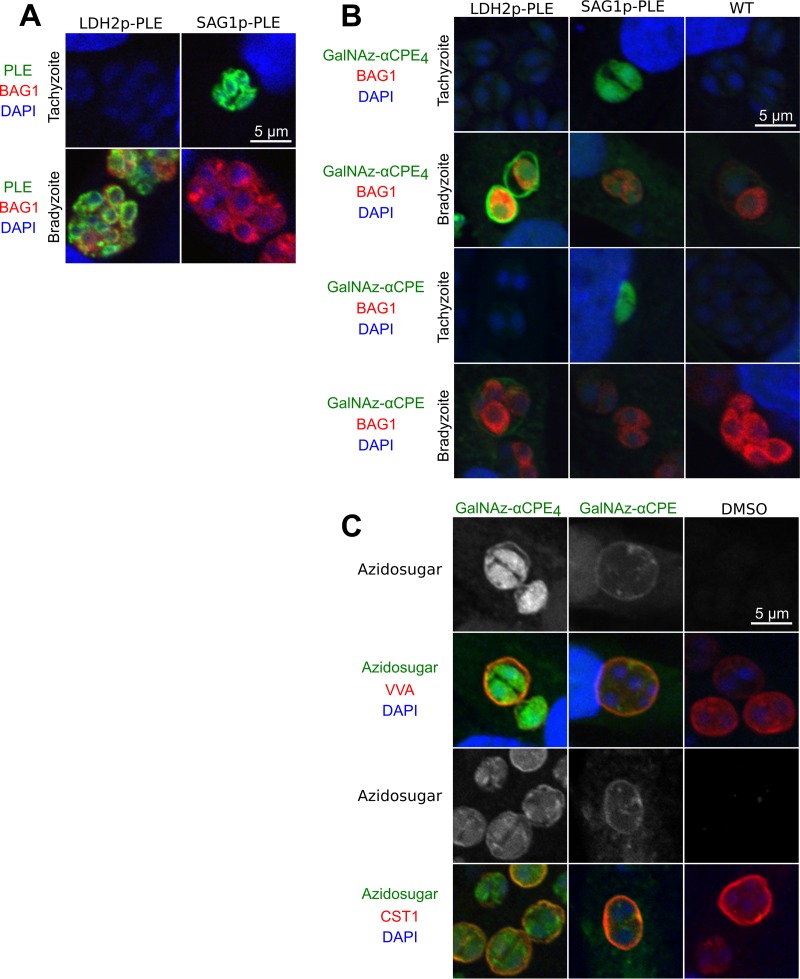
Stage-specific sugar labeling with esterase-ester click chemistry. (A) IFA of intracellular parasites expressing PLE (green) under either LDH2 (bradyzoite-specific) and SAG1 (tachyzoite-specific) promoters cultured under tachyzoite (pH 7) or bradyzoite (pH 8) conditions. The parasite’s stage differentiation was monitored by using the bradyzoite marker BAG1 (red). PLE is stage specifically expressed. (B) IFA of intracellular parasites incubated with caged azidosugars (green) under tachyzoite and bradyzoite conditions. Uncaging and sugar labeling is stage specifically achieved. (C) Costaining of azidosugars (GalNAz-αCPE4 or GalNAz-αCPE, green) and *O*-GalNAc-specific lectin VVA (red) on the intracellular T. gondii. VVA and azidosugar colabels parasitophorous cyst walls. Another *O*-GalNAc-specific monoclonal antibody against CST1 also colabels with azidosugars (lower panels).

The advantage of using a genetically encoded enzyme for uncaging a small chemical is that it allows temporal regulation of enzyme expression by altering its promoter. We evaluated the ability of PLE to be expressed in a stage-specific fashion and to selectively label stage-specific glycans. Previous data have shown that lectin binding pattern changes during parasite differentiation from tachyzoites to bradyzoites. To label parasite glycans in stage-specific manner, we used, in place of the constitutive promoter from GRA1, two well-characterized stage-specific promoters: (i) the bradyzoite-specific promoter lactate dehydrogenase 2 (LDH2), and (ii) the tachyzoite-specific promoter SAG1 to drive PLE expression. Figure 4A demonstrates that parasites express the PLE stage specifically according to their stage-specific promoters. In addition, this also demonstrates that PLE degrades rapidly enough that there is a minimal leak in these stage-specific promoter constructs into the other life cycle stage. The stage-specific expression of the PLE using LDH2 and SAG1 promoters allowed PLE to selectively uncage GalNAz-αCPE_4_ in bradyzoites or tachyzoites, respectively (Fig. 4B, upper two rows). Similarly, GalNAz-αCPE was also stage specifically uncaged though at a lower efficiency (Fig. 4B, lower two rows).

T. gondii generates a cyst wall containing *O*-GalNAc glycosylated proteins. To investigate whether the azidosugar signal colocalized with the known *O*-linked GalNAc markers, we stained these parasites with *Vicia villosa* lectin (VVA), which is specific to α or β1,4-GalNAc-O-Ser/Thr (15). Figure 4C shows the results of immunofluorescence analyses (IFA) of intracellular parasites, under bradyzoite culture conditions, incubated with either GalNAz-αCPE_4_ or GalNAz-αCPE. VVA stains the cyst wall and the GalNAz-αCPE_4_ partially colocalizes with additional intracellular staining. On the other hand, GalNAz-αCPE only stains the cyst wall, although in low intensity. Azidosugar-treated parasites were also costained with an antibody specific to the known *O*-GalNAc modified cyst wall protein CST1. The costaining displayed a similar staining pattern for the cyst wall and the azidosugar signal, suggesting that these azidosugars are probably incorporated into the cyst wall glycoproteins.

## DISCUSSION

In this proof-of-concept study, we demonstrate the selective delivery of caged sugars using a combination of an esterase-ester pair system and bioorthogonal click chemistry. This method provides a robust system for the delivery of small molecules to the intracellular parasite T. gondii, while eliminating host cell labeling. In addition, this system can provide stage-specific delivery of small molecules. This is supported by our data that demonstrates liberation of the azidosugars being observed only in PLE-expressing intracellular parasite, but not in the surrounding host cells, and by our ability to selectively label either tachyzoite- or bradyzoite-stage parasite glycans using stage-specific promoter PLE constructs. This provides a method to deliver caged molecules to an intracellular parasite without affecting surrounding host cells and to deliver molecules to specific types of cells in heterogeneous populations by selective expression of this genetically encoded uncaging enzyme. The simple and robust combination of esterase-ester pair system with click chemistry can be readily applied to other small molecule inhibitors or metabolic substrates that can be modified with the cyclopropyl ester. For example, instead of labeling the nascent protein in all heterogeneous cell types in the puromycin-associated nascent chain proteomics (PUNCH-P) (16) metabolic labeling system, we could selectively label specific cell types by the expression of PLE similar to other cell-selective noncanonical amino acid incorporation methods using the DDC/Lyr system (17), the PhAc-OP-puro system (18), the MetRS-BONCAT system (19), and the eNTR-NMOG system (20).

Azide signal was present in cyst matrix and cyst wall in bradyzoite vacuoles, suggesting that cyst-specific *O*-GalNAc glycans are the likely source of this staining. Azide signal is also seen in the nucleus of PLE-expressing tachyzoites (Fig. 3). *O*-GalNAc glycosylation is confined in the secretory pathway, and a recent comprehensive mass spectrometry glycomics study demonstrated that T. gondii is devoid of any *O*-GlcNAc glycosylation (5). In order to determine what components are labeled by the azide moiety, further experiments, such as click chemistry affinity purification, are needed. The PLE method and reagents can be used to isolate labeled glycans/glycoproteins and to subsequently identify glycoproteins, the site of modification, and glycans by mass spectrometry. These types of studies would provide new insights into secreted glycoproteins of intracellular parasites.

## MATERIALS AND METHODS

### Construction of PLE-expressing *T. gondii*.

To express PLE constitutively in T. gondii in ER, a plasmid construct was made by concatenating GRA1 promoter (TGME49_270250), PLE signal peptide, FLAG tag sequence, the rest of PLE (11) with the ER retention signal swapped from the original HAEL to the T. gondii BiP retention signal HDEL (14), and a mutant dihydrofolate reductase (21) selectable marker containing vector using an NEBuilder HiFi DNA assembly kit (NEB). The plasmid pBS-GRA1p-PLE-FLAG-DHFR sequence and the primers used for construction are presented in Text S2 in the supplemental material. The linearized plasmid (SwaI; NEB) was transfected into the type II ME49 strain of parasites with intact *KU80* gene by electroporation. Parasites were cultured in the presence of a selectable drug pyrimethamine at 1 μM for 3 days immediately after the transfection. Subsequently, the parasites were subcloned by limiting dilution with 96-well plate, and clones were screened by diagnostic PCR. The expression of PLE in the parasites were confirmed by IFA using FLAG-specific antibody (Sigma).

10.1128/mSphere.00142-19.2 TEXT S2 pBS-GRA1p-PLE-FLAG-DHFR sequence (GenBank format). Download Text S2, TXT file, 0.02 MB. Copyright © 2019 Tomita et al. 2019 Tomita et al. This content is distributed under the terms of the Creative Commons Attribution 4.0 International license.

To express PLE in a stage-specific fashion, the GRA1 promoter of the aforementioned plasmid construct was replaced with either SAG1 promoter (tachyzoite specific, TGME49_233460), LDH2 promoter (bradyzoite specific, TGME49_291040), or BAG1 promoter (bradyzoite specific, TGME49_259020). The plasmid sequences and primers used for the DNA assembly are presented in Text S1 in the supplemental material. ME49 parasites were transfected and cloned as described above.

### Immunofluorescent assay for PLE expression in *T. gondii*.

Human foreskin fibroblasts (HFFs; ATCC CRL-1634; Hs27), grown on circular cover glass (no. 1.5H, 12-mm circle), were infected with either PLE-expressing or wild-type parasites for 5 days. Cells on the cover glass were fixed with 4% paraformaldehyde in phosphate-buffered saline (PBS) for 30 min and then permeabilized with 0.2% Triton X-100 for 20 min at room temperature. After permeabilization, the samples were blocked with 1% bovine serum albumin (BSA) in PBS at room temperature for 1 h. The cover glasses were then incubated with mouse anti-FLAG monoclonal antibody M2 (Sigma) diluted at 1:200 and rabbit anti-T. gondii polyclonal antibody diluted at 1:1,000 at 37°C for 1 h. The cover glasses were then washed three times with 0.1% BSA in PBS, followed by incubation with anti-mouse–Alexa Fluor 594 and anti-rabbit–Alexa Fluor 488 antibodies for 1 h at 37°C. The cover glasses were then washed twice with PBS and mounted using ProLong Gold antifade (Thermo Fisher).

### FITC-CM_2_ cleavage assay.

HFFs, grown in a two-well glass chamber, were infected with either PLE-expressing or wild-type parasites for 4 days. Medium was replaced with 10% FBS in PBS containing fluorescein di(1-methylcyclopropanecarboxymethyl ester) substrate (FITC-CM_2_) at 10 μM (a generous gift from Luke D. Lavis [11]). After a 30-min incubation at 37°C, the medium was replaced with 10% FBS in PBS. Images were taken immediately with Diaphot 300 epifluorescence microscope (Nikon) with equal exposure times between wild-type and PLE-expressing parasites.

### Visualization of azidosugars with click chemistry.

HFFs, grown on circular cover glass no. 1.5, were infected with either PLE-expressing or wild-type parasites for a day. The medium was replaced with complete medium with azidosugars or dimethyl sulfoxide. After a 2-day incubation with the azidosugars, the cells on a cover glass were fixed with 4% paraformaldehyde in PBS for 30 min and then permeabilized with 0.2% Triton X-100 for 20 min. The cells were incubated in 50 μM CuSO_4_, 100 μM 3-[4-({bis[(1-*tert*-butyl-1H-1,2,3-triazol-4-yl)methyl]amino}methyl)-1*H*-1,2,3-triazol-1-yl]propanol (BTTP) (22), 50 μM Alexa Fluor 488–alkyne (Life Technologies), 2.5 mM sodium ascorbate, and 1% FBS in PBS for 20 min at room temperature. The cells were subsequently washed and immunostained with rabbit polyclonal anti-BAG1 antibody at 1:500, anti-CST1 antibody at 1:500, or biotinylated VVA lectin (Vector Laboratories) at 1:100 for 1 h at room temperature. The antibodies and the lectin were visualized by incubation with goat anti-rabbit–Alexa Flour 594 antibody at 1:2,000 or streptavidin-594 at 1:2,000 for 1 h at room temperature. The cover glass was then mounted on glass slides with the antifade.
