# Supplementary material for: Stage-Specific and Selective Delivery of Caged Azidosugars into the Intracellular Parasite Toxoplasma gondii by Using an Esterase-Ester Pair Technique
Source: mSphere. 2019 May 29;4(3):e00142-19. doi: 10.1128/mSphere.00142-19 (PMC6541734; doi:10.1128/mSphere.00142-19)
Supplement: TEXT S1 [file mSphere.00142-19-s0001.pdf]

Supplemental Materials: Method S1.

Synthesis of caged azidosugars

Scheme 1: Synthesis of probe **4** (GalNAz-αCPE)

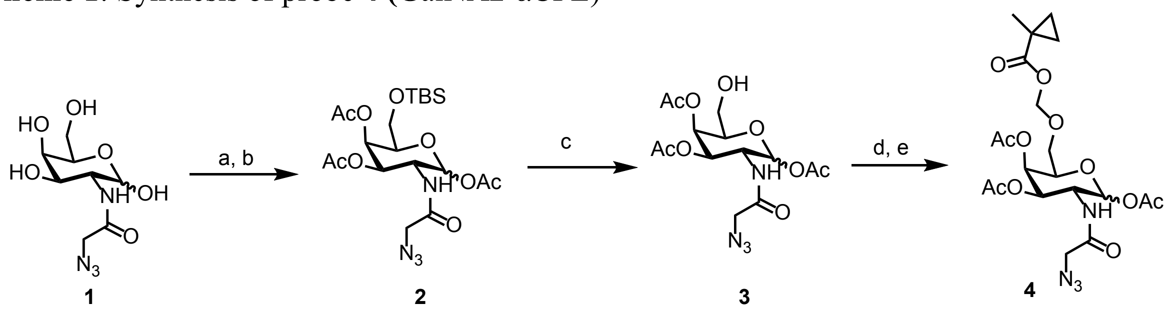

a: TBSCl (1.5 equiv.), DMAP (0.05 equiv.), pyridine, rt, 24 h; then b: Ac_2O, rt, 24 h, yield (2 steps): 55%; c: TBAF (1.1 equiv.) HOAc/TFA/water, 24 h, yield: 71%; d: (chloromethyl)(methyl)sulfane (1.5 equiv.), $AgNO_3$ (1.5 equiv.), Et_3N (1.5 equiv.), Toluene/DCM 60 °C, 4 h; then e: SO_2Cl_2 (2 equiv.), DCM, rt, 3 h, 1-methylcyclopropane-1-carboxylic acid (2 equiv.), Et_3N (2 equiv.), THF, yield (two steps): 6%.

To a well-stirred solution of 2-[(2-azidoacetyl)amino]-2-deoxy-D-galactose **1** (793 mg, 3.02 mmol) in pyridine (20 mL) was added TBSCl (684 mg, 4.54 mmol) and DMAP (18.5 mg, 0.15 mmol). The reaction mixture was allowed to stir at room temperature for 24 hours, and then acetic anhydride (10 mL, 91 mmol) was added dropwise. The mixture was stirred at room temperature for another 24 hours, concentrated under vacuum, and purified by silica gel column chromatography (EA/Hex 1/5, 1/4, 1/3, 1/2) to afford 2-[(2-azidoacetyl)amino]-2-deoxy-6-(tert-butyldimethylsilyloxyl)-6-deoxy-D-galactose-1,3,4-triyl triacetate **2** (829 mg, α/β = 1/5 yield: 55%). β conformation of compound **2**: ^1H NMR (300 MHz, $CDCl_3$) δ 6.32 (d, J = 9.2 Hz, 1H), 6.22 (d, J = 3.9 Hz, 1H), 5.54 (d, J = 3.2 Hz, 1H), 5.28 (dd, J = 11.6, 3.3 Hz, 1H), 4.68 (ddd, J = 12.2, 9.2, 3.6 Hz, 1H), 4.10 – 4.02 (m, 1H), 4.00 – 3.89 (m, 3H), 3.71 – 3.50 (m, 3H), 2.19 (s, 3H), 2.16 (s, 3H), 2.03 (s, 3H), 0.86 (s, 12H), 0.02 (d, J = 3.2 Hz, 6H); ^{13}C NMR (75 MHz, $CDCl_3$) δ 171.1, 170.6, 169.9, 169.1, 167.0, 91.1, 71.4, 68.1, 66.6, 60.5, 52.6, 47.3, 25.8, 21.0, 20.8, 20.7, -5.6, -5.7; MS (ESI) [M+Na] 525.3.

To a solution of 2-[(2-azidoacetyl)amino]-2-deoxy-6-(tert-butyldimethylsilyloxyl)-6-deoxy-D-galactose-1,3,4-triyl triacetate **2** (120 mg, 0.24 mmol) in a mixture of HOAc (3 mL), THF (1 mL),

water (1 mL) was added TBAF (1 M in THF, 0.26 mL, 0.26 mmol). The mixture was allowed to stir at room temperature for 6 hours, neutralized by Sat. $NaHCO_3$ (10 mL), then extracted 3 times with ethyl acetate (50 mL). The organic phase was combined, dried over Na_2SO_4, concentrated under vacuum and the crude product was purified by silica gel column chromatography ((EA/Hex 1/1, Acetone/Hex 1/2, then Acetone/Hex 1/1) to afford 2-[(2-azidoacetyl)amino]-2-deoxy-D-galactose-1,3,4-triacetate **3** (66 mg, yield: 71%). β conformation of compound **3**: ^{1}H NMR (300 MHz, $CDCl_3$) δ 6.45 (d, J = 9.1 Hz, 1H), 6.22 (d, J = 3.8 Hz, 1H), 5.44 (d, J = 3.1 Hz, 1H), 5.32 – 5.25 (m, 1H), 4.71 (ddd, J = 11.4, 9.1, 3.9 Hz, 1H), 4.10 (t, J = 6.5 Hz, 2H), 3.99 – 3.88 (m, 2H), 3.58 (ddd, J = 51.8, 11.8, 6.5 Hz, 2H), 2.20 (s, 3H), 2.19 (s, 3H), 2.05 (s, 3H); ^{13}C NMR (75 MHz, $CDCl_3$) δ 171.2, 171.1, 169.3, 167.3, 91.0, 71.6, 67.8, 67.6, 60.7, 52.5, 47.4, 21.0, 20.8, 20.7. MS (ESI) [M+Na] 411.3.

To a stirred solution of $AgNO_3$ (227 mg, 1.34 mmol), triethylamine (135 mg, 1.34 mmol) in toluene (2 mL) was added 2-[(2-azidoacetyl)amino]-2-deoxy-D-galactose-1,3,4-triacetate **3** and (346 mg, 0.891 mmol) and (chloromethyl)(methyl)sulfane (129 mg, 1.34 mmol) in CH_2Cl_2 (2 mL) dropwise at room temperature. The mixture was then heated to 60 °C overnight, diluted with toluene (10 mL), filtered through celite, and washed with toluene (30 mL). The organic was combined, concentrated over vacuum, and briefly purified by silica gel column chromatography (EA/Hex 1/4, 3/1, 2/1, 1/1) to afford crude 2-[(2-azidoacetyl)]-6-(methylthiomethoxy)-6-deoxy-D-galactosamine-1,3,4-triacetate 60 mg which was then dissolved in DCM (5 mL) for next step without further purification. Sulfuryl dichloride (1 M in DCM, 270 uL) was added dropwise to the stirred solution of 2-[(2-azidoacetyl)]-6-(methylthiomethoxy)-6-deoxy-D-galactosamine-1,3,4-triacetate in DCM and the mixture was allowed to stir at room temperature for 3 hours. DCM was then distilled under vacuum and the residue was re dissolved in THF (5 mL), followed by addition of 1-methylcyclopropane-1-carboxylic acid (26.8 mg, 0.27 mmol) and triethylamine (27.1 mg, 0.27 mmol). The mixture was then allowed to stir at room temperature overnight, diluted with ethyl

acetate (50 mL), washed with 0.1 M HCl (30 mL), sat. $NaHCO_3$ (20 mL). The organic phase was dried over Na_2SO_4, concentrated, and the residue was purified by silica gel column chromatography (EA/Hex 1/4, 1/3, 1/2, 1/1) to afford 2-[(2-azidoacetyl)amino]-2-deoxy-6-[(1-methylcyclopropane-1-carbonyl)oxy)methoxy)]-6-deoxy-D-galactose-1,3,4-triacetate **4** (25 mg, two step yield: 6%). β conformation of compound **4**: ^{1}H NMR (300 MHz, Chloroform-*d*) δ 6.32 (d, *J* = 9.1 Hz, 1H), 6.24 (d, *J* = 3.7 Hz, 1H), 5.50 (d, *J* = 3.1 Hz, 1H), 5.30 – 5.13 (m, 3H), 4.71 (ddd, *J* = 12.2, 9.2, 3.8 Hz, 1H), 4.19 (t, *J* = 6.3 Hz, 1H), 3.95 (s, 2H), 3.71-3.57 (m, 2H), 2.20 (s, 3H), 2.19 (s, 3H), 2.04 (s, 3H), 1.31 (s, 3H), 1.29 – 1.22 (m, 2H), 0.77-0.70 (m, 2H); ^{13}C NMR (75 MHz, $CDCl_3$) δ 175.4, 171.0, 170.1, 169.0, 167.0, 91.0, 89.2, 69.9, 67.8, 67.7, 67.1, 52.5, 47.2, 21.0, 20.8, 20.7, 19.3, 18.6, 17.2, 17.2. MS: (ESI) [M+Na] 523.2. HRMS: (ESI) $C_{20}H_{28}N_4NaO_{11}$: calc. 523.1652; found 523.1659.

To a well stirred solution of 2-[(2-azidoacetyl)amino]-2-deoxy-D-galactose **1** (63 mg, 0.24 mmol), Et_3N (242 mg, 2.4 mmol) in DCM (5 mL) was added 1-methylcyclopropane-1-carbonyl chloride

Scheme 2: Synthesis of probe **6** (GalNAz-αCPE$_4$)

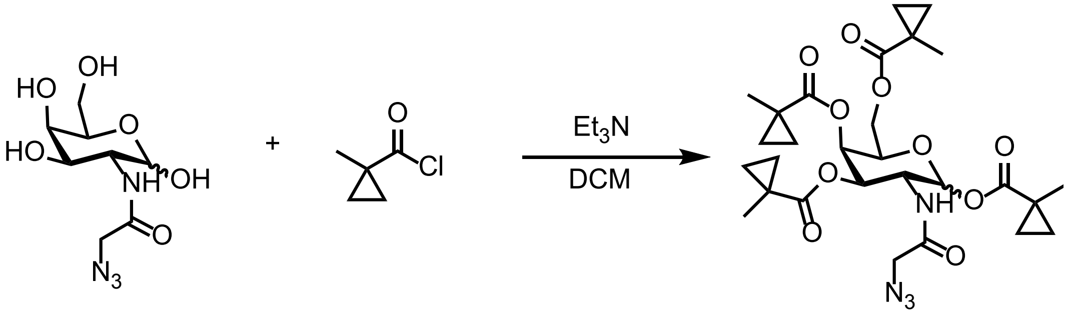

1

21%

6

(283 mg, 2.4 mmol) in 5 minutes at 0 °C. The mixture was allowed to stir at room temperature for 48 hours, and then diluted with ethyl acetate (30 mL). The organic was washed with HCl (1M, 30 mL), sat. $NaHCO_3$ (30 mL), water (30 mL), brine (20 mL), concentrated and purified by silica gel column chromatography to afford 2-[(2-azidoacetyl)amino]-2-deoxy-D-galactose-1,3,4,5-tetra[1-

methylcyclopropanecarboxylate] **6** (30 mg, α/β ≈ 1/1; yield: 21%). Mixture of α and β conformation of compound **4**: ^{1}H NMR (600 MHz, $CDCl_3$) δ 6.91 (d, *J* = 7.8 Hz, 1H), 6.35 (d, *J* = 8.9 Hz, 1H), 6.24 (d, *J* = 4.9 Hz, 1H), 6.21 (d, *J* = 3.6 Hz, 1H), 5.47 (d, *J* = 3.1 Hz, 1H), 5.32 (dd, *J* = 8.5, 7.2 Hz, 1H), 5.24 (dd, *J* = 11.4, 3.2 Hz, 1H), 5.17 (td, *J* = 6.4, 3.6 Hz, 1H), 4.66 (td, *J* = 8.1, 4.9 Hz, 1H), 4.59 (ddd, *J* = 12.0, 9.1, 3.6 Hz, 1H), 4.32 (dd, *J* = 12.1, 3.7 Hz, 1H), 4.22 (m, 2H), 4.13 – 4.05 (m, 2H), 4.04 – 4.00 (m, 1H), 3.98 (d, *J* = 4.0 Hz, 2H), 3.95 (d, *J* = 8.7 Hz, 2H), 1.50 – 1.11 (m, 40H), 0.93 – 0.62 (m, 16H). ^{13}C NMR (151 MHz, $CDCl_3$) δ 175.7, 175.3, 174.9, 174.8, 174.3, 174.2, 173.5, 173.4, 166.4, 166.3, 93.6, 90.5, 78.3, 73.7, 69.9, 68.5, 67.5, 66.2, 62.0, 61.7, 601.0, 56.1, 52.1, 52.0, 47.4, 18.9, 18.8, 18.7, 18.7, 18.7, 18.6, 18.5, 18.4, 18.2, 18.2, 18.1, 18.0, 18.0, 18.0, 18.0, 17.3, 17.2, 17.1, 17.1, 17.1, 17.0, 16.9, 16.9, 16.8, 16.6, 16.6, 16.6, 16.6, 16.4. HRMS: (ESI) $C_{28}H_{38}N_4NaO_{10}$: calc. 613.2486; found 613.2480.
